# Supplementary material for: Machine learning models to accelerate the design of polymeric long-acting injectables
Source: Nat Commun. 2023 Jan 10;14:35. doi: 10.1038/s41467-022-35343-w (PMC9832011; doi:10.1038/s41467-022-35343-w)
Supplement: Supplementary file 1 — Supplementary Information [file 41467_2022_35343_MOESM1_ESM.pdf]

Supplementary Information

Machine Learning Models to Accelerate the Design of Polymeric Long-Acting Injectables

Pauric Bannigan[1], Zeqing Bao[1], Riley J. Hickman[2,3,4], Matteo Aldeghi[2,3,4], Florian Häse[2,3,4], Alán Aspuru-Guzik[2,3,4,5,6,7*], Christine Allen[1*]

[1]Leslie Dan Faculty of Pharmacy, University of Toronto, Toronto, ON M5S 3M2, Canada.

[2]Department of Computer Science, University of Toronto, Toronto, ON M5S 3H6, Canada.

[3]Department of Chemical Engineering & Applied Chemistry, University of Toronto, Toronto, ON M5S 3E5, Canada

[4]Vector Institute for Artificial Intelligence, Toronto, ON M5S 1M1, Canada.

[5]Department of Materials Science & Engineering, University of Toronto, Toronto, ON M5S 3E4, Canada

[6]Lebovic Fellow, Canadian Institute for Advanced Research, Toronto ON, M5S 1M1, Canada.

[7]CIFAR Artificial Intelligence Research Chair, Vector Institute, Toronto, ON, M5S 1M1, Canada

These authors jointly supervised this work: Alán Aspuru-Guzik, Christine Allen

* Corresponding authors

*Christine Allen
Leslie Dan Faculty of Pharmacy,
University of Toronto,
Toronto, ON, M5S3M2,
Canada
Email: cj.allen@utoronto.ca

*Alan Aspuru-Guzik
Department of Chemistry,
University of Toronto,
Toronto, ON, M5S 3H6
Canada
E-mail: alan@aspuru.com

Supplementary Figures:

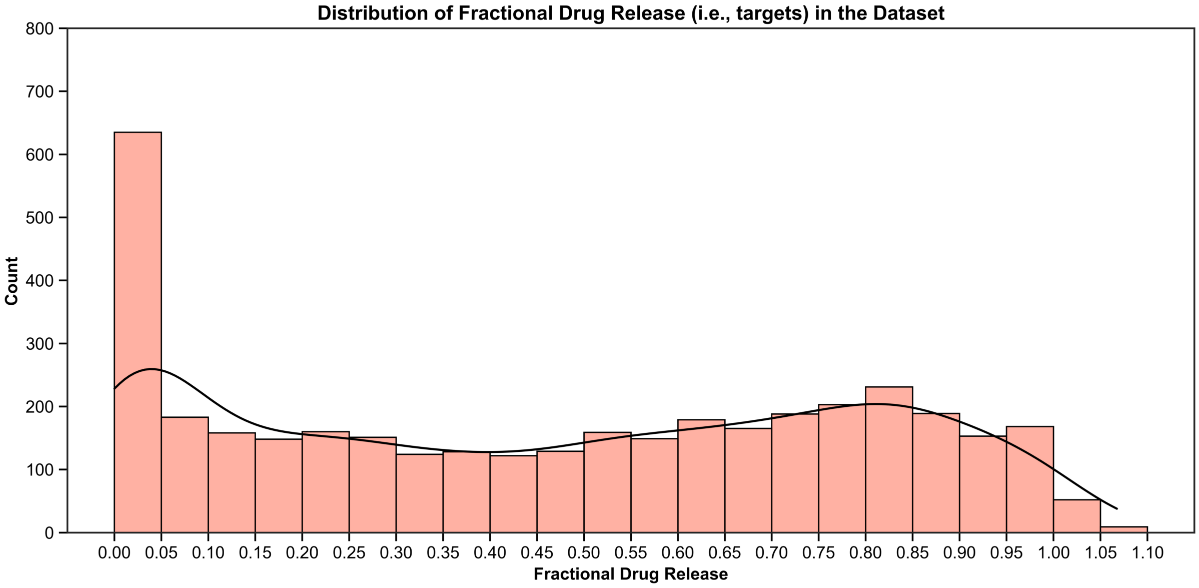

Supplementary Figure 1: **Distribution of the fractional drug release values for the 17-feature datasets arranged into bins with increments of 0.05.**

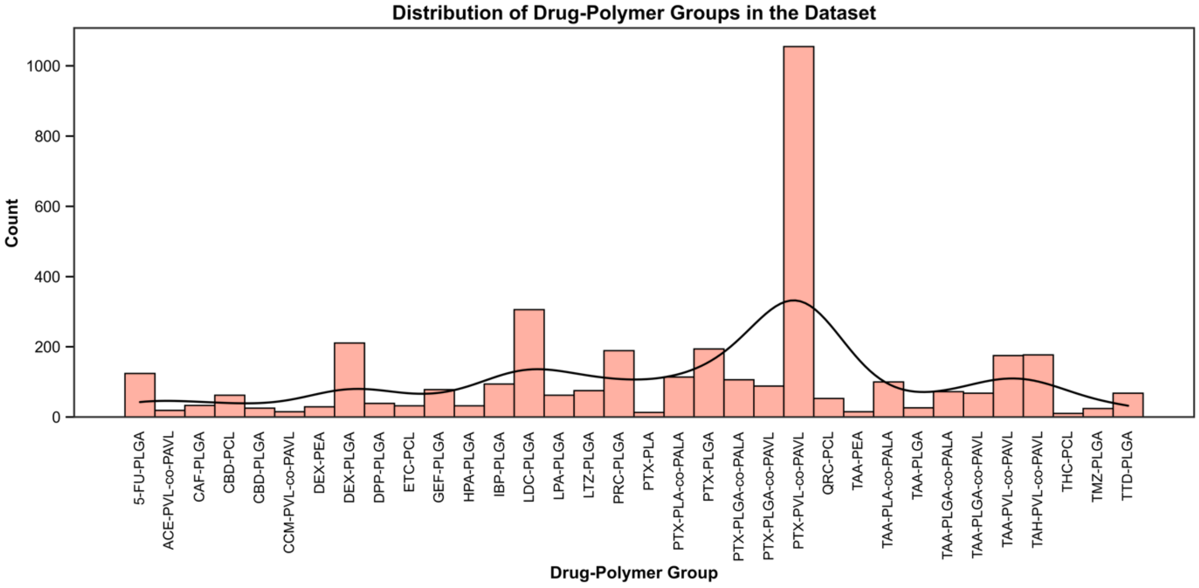

Supplementary Figure 2: **Distribution of drug-polymer groups in the 17-feature dataset.**

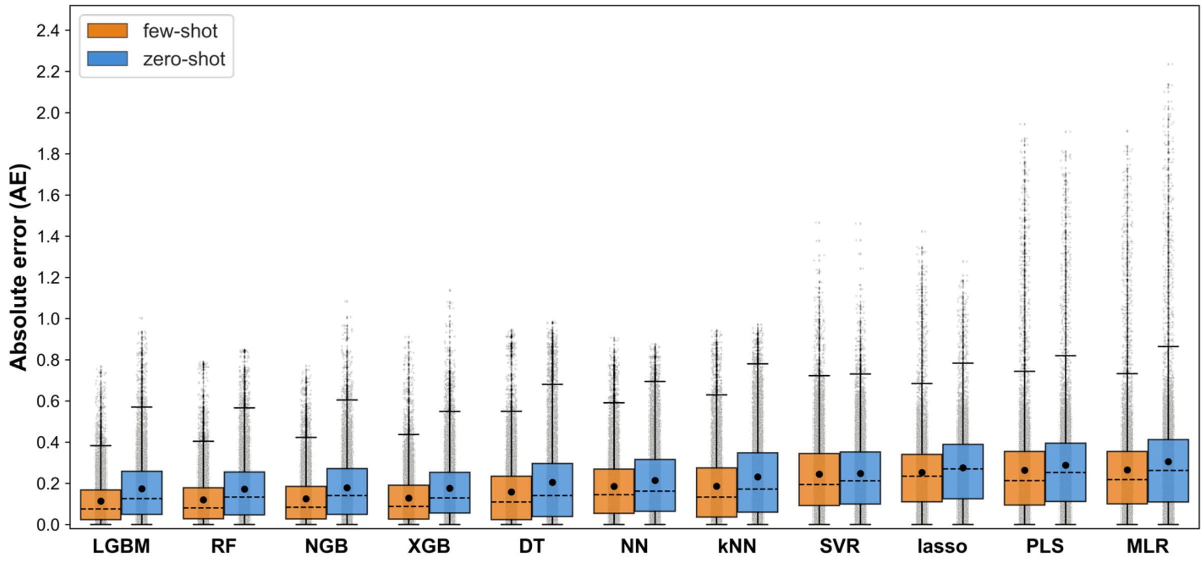

Supplementary Figure 3. **Summary of the overall predictive performance of the various ML models represented as a box and whisker plot (with and without initial drug release timepoints).** Few-shot learners (i.e., including three initial drug release datapoints) are shown as orange boxplots. Zero-shot learners (i.e., not including any initial drug release datapoints) are shown as blue boxplots. The data represent the absolute error (AE) obtained for fractional drug release predictions during nested cross-validation (i.e., n = 10 trials). Each column represents the AE value for 8013 data instances from the collective nested cross-validation test sets (light grey circles). The mean absolute error (MAE) and median AE values for each model are displayed within the boxplots as closed black circles and black dashed lines, respectively. The first and second quartile are shown by the upper and lower edges of the respective boxes. The whiskers extend to show the rest of the distribution, excluding points determined to be "outliers" using the inter-quartile range method. Source data are provided as a Source Data file.

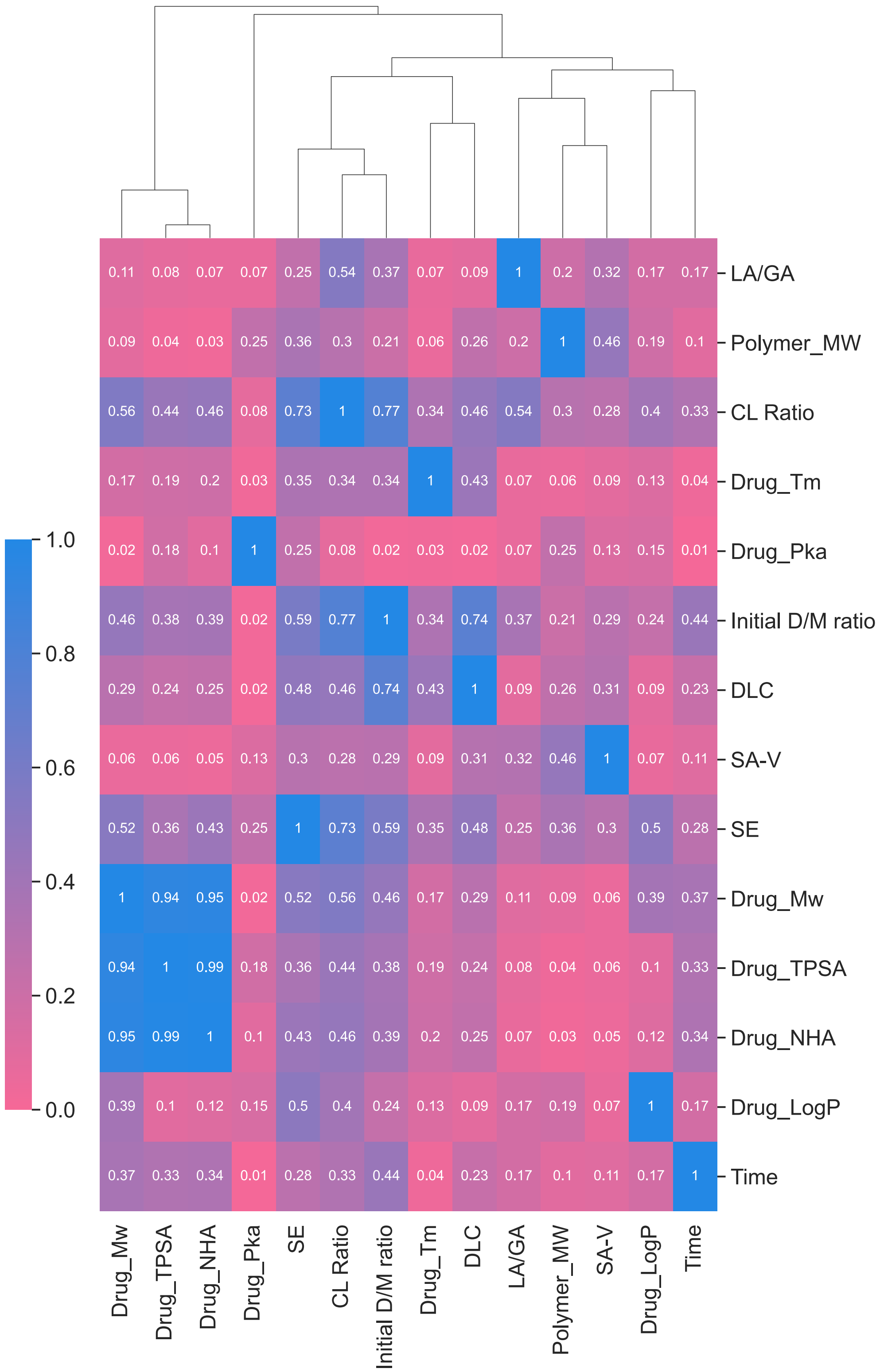

Supplementary Figure 4. **Model refinement of the zero-shot RF model (i.e., most accurate zero-shot model).** Heatmap of the absolute Spearman's Rank correlation between the initial 14 input features with dark blue signifying an absolute Spearman's Rank correlation = 1 and white representing an absolute Spearman's Rank correlation of 0. Attached to the heatmap is a dendrogram which displays the hierarchies of feature clusters that were determined via agglomerative hierarchical clustering analysis. Source data are provided as a Source Data file.

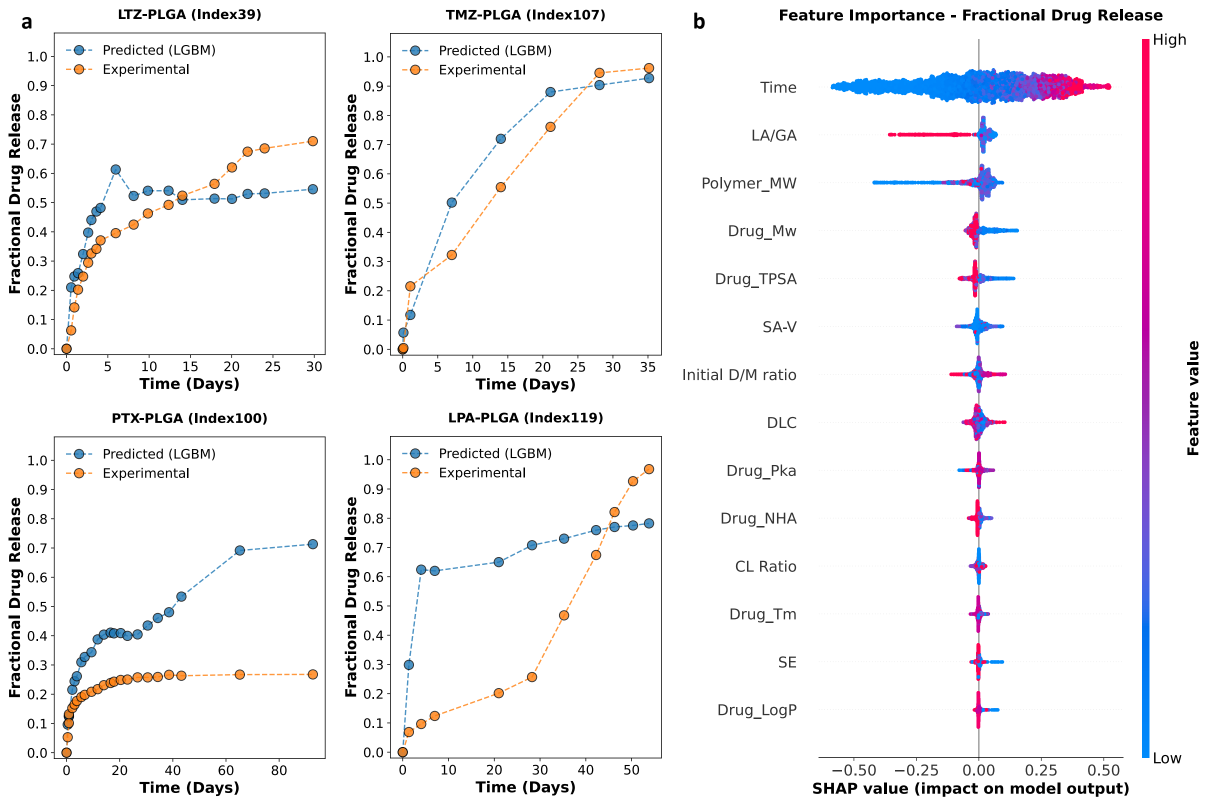

Supplementary Figure 5. **Model interpretation of the zero-shot RF model (i.e., most accurate zero-shot model). (a)** Select examples of experimental fractional drug release profiles (orange circles) in comparison to predicted fractional drug release profiles (blue circles) generated by the lightGBM model (LGBM). **(b)** Shapley additive explanations (SHAP) analysis for the 14-feature RF. The impact of each feature on fractional drug release is illustrated through a swarm plot of their corresponding SHAP values. The color of the dot represents the relative value of the feature in the dataset (high-to-low depicted as pink-to-blue). The horizontal location of the dots shows whether the effect of that feature value contributed positively or negatively in that prediction instance (x-axis). Source data are provided as a Source Data file.

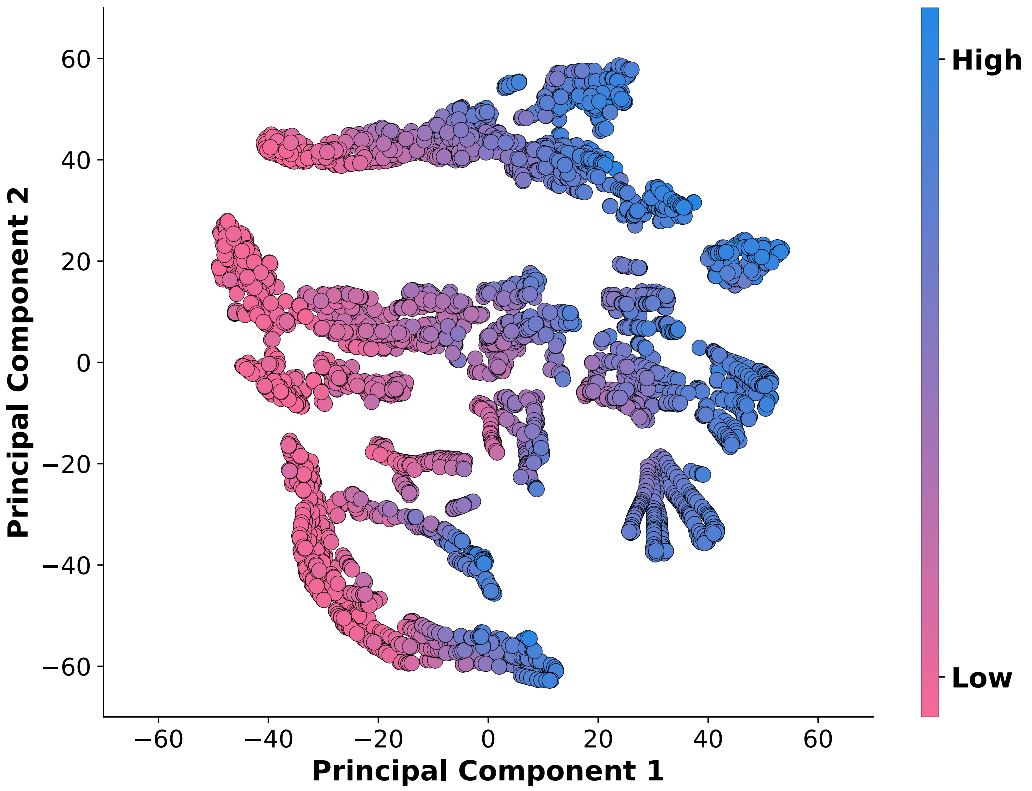

Supplementary Figure 6: Two-dimensional visualization of the SHAP values calculated for the input features of the lightGBM model. The SHAP values for the 15 input features were condensed into two principal components using principal component analysis (PCA) and then grouped together using a simple unsupervised clustering algorithm (T-distributed Stochastic Neighbor Embedding). The attached colorbar indicates the relative SHAP values in the dataset ranked from high (blue) to low (pink).

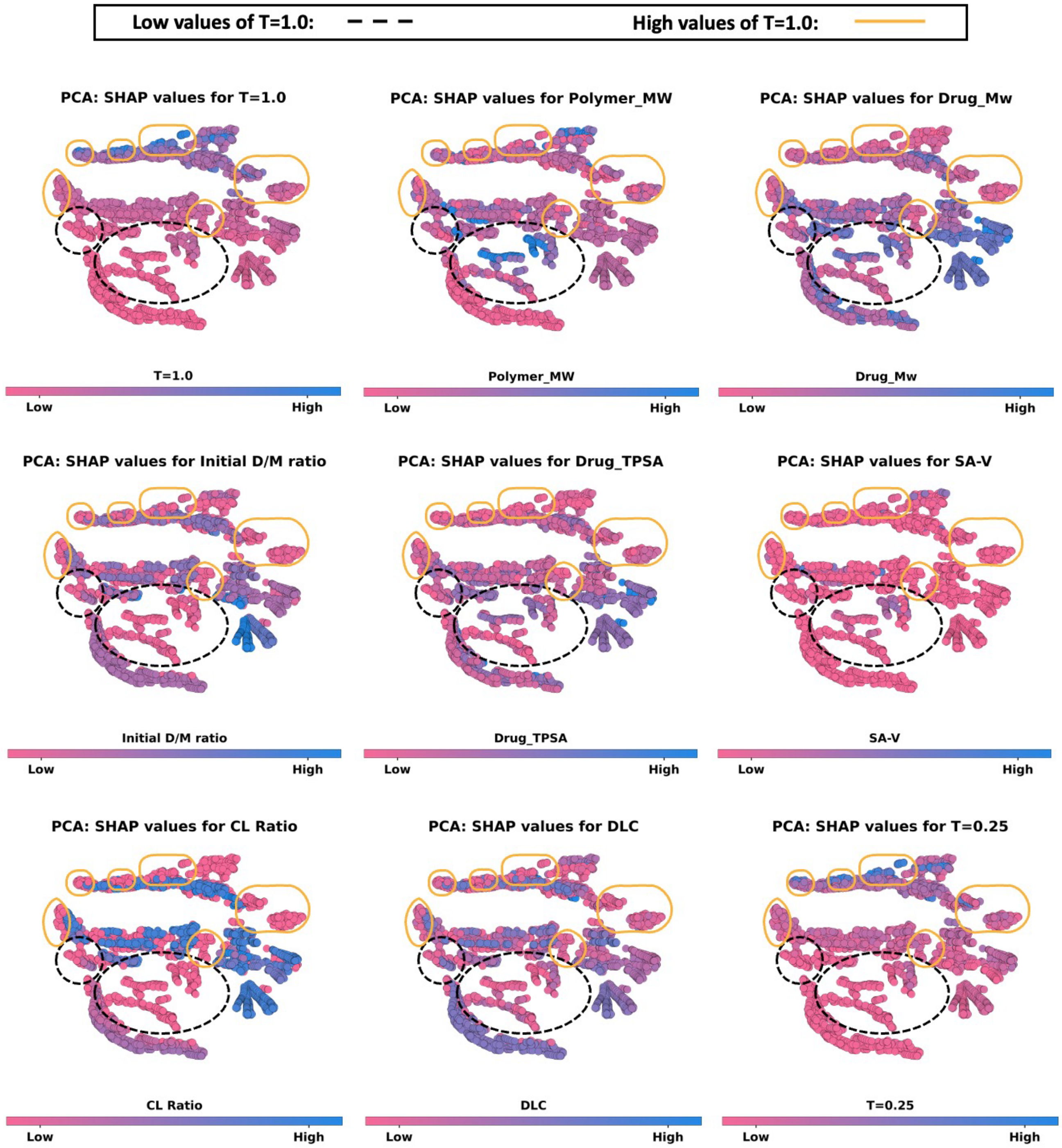

Supplementary Figure 7: Two-dimensional visualization of the SHAP values calculated for the input features of the lightGBM model. The SHAP values for the 15 input features were condensed into two principal components using principal component analysis (PCA) and then grouped together using a simple supervised clustering algorithm (T-distributed Stochastic Neighbor Embedding). The attached color bars indicate the relative SHAP values in the dataset ranked from high (blue) to low (pink). Each of the subplots allows for visualization and comparison of the location of data instances corresponding to different input features. The locations of clusters corresponding to non-crosslinked systems with high initial release (i.e., high values of *T=1.0*) and low initial release (i.e., low values of *T=1.0*) are highlighted as black and orange lines, respectively. Source data are provided as a Source Data file.

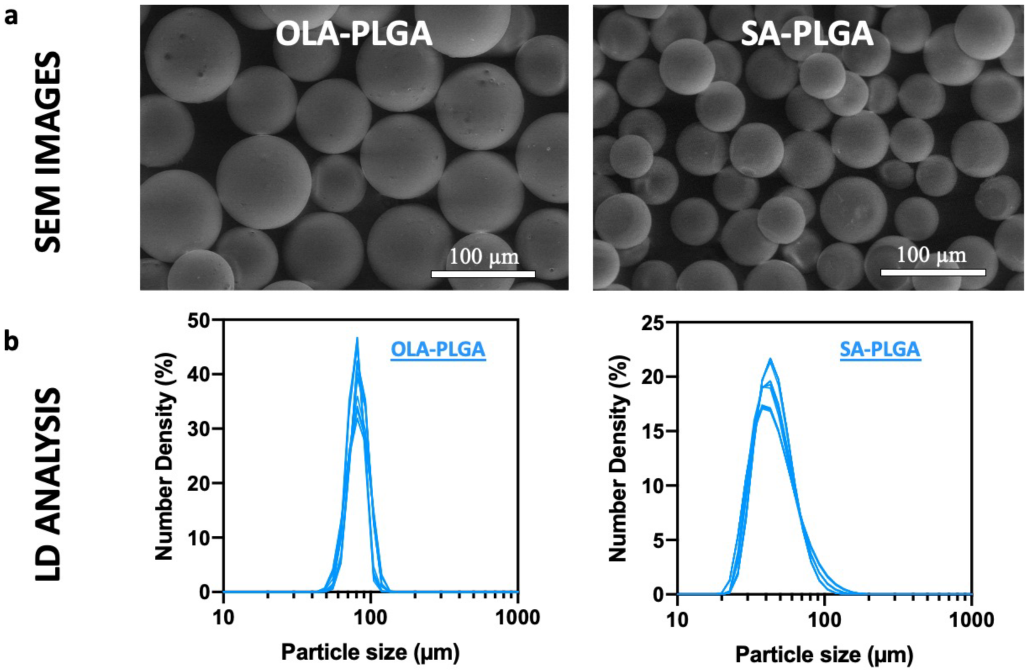

Supplementary Figure 8. Summary of the particle size analysis conducted on the olapirib-PLGA microparticles (OLA-PLGA) and salicylic acid-PLGA microparticles (SA-PLGA). (a) Representative scanning electron microscopy (SEM) images of OLA-PLGA and SA-PLGA illustrating the morphology of the drug loaded microparticles. **(b)** Size and size distribution of the OLA-PLGA and SA-PLGA microparticles as determined by laser diffraction (LD) particle size analysis.

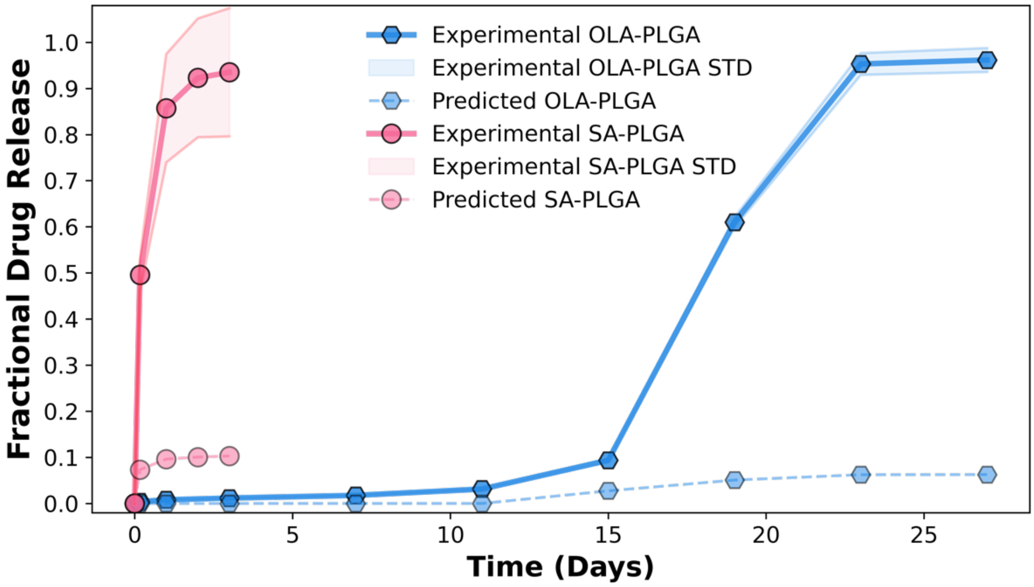

Supplementary Figure 9. **Prospective study results for the zero-shot RF model (i.e., most accurate zero-shot model).** Comparison of the experimental and predicted fractional drug release profiles for the salicylic acid-PLGA MP (SA-PLGA) and olaparib-PLGA MP (OLA-PLGA) formulations. Three independent batches of both formulations were prepared, and their experimental drug release was characterized in 0.5 wt% sodium dodecyl sulfate (SDS) to ensure sink conditions throughout the experiments. The experimental fractional drug release profiles for SA-PLGA and OLA-PLGA are plotted together as dark red circles with a solid line (SA-PLGA) and dark green hexagons with a solid line (OLA-PLGA), respectively. In both cases the standard deviation (STD) observed for the experimental drug release measurements is displayed as a coloured halo (*n*=3). The fractional drug release profiles predicted by the zero-shot RF model are also shown as pale red circles with a broken line (SA-PLGA) and pale green hexagons with a broken line (OLA-PLGA), respectively. Source data are provided as a Source Data file.

Supplementary Tables:

Supplementary Table 1: Table illustrating how an LAI system (PLGA-; Experimental index 80) is described to the various ML models as a series of input features (i.e., 17 features). Highlighted (in yellow) are the values for drug release time points (Time), which are the only input features that vary for a given LAI system. Units of Fraction Drug Release are abbreviated as "FRD".

LA/GA	Polymer MW (Da)	CL Ratio	Drug Tm (°C)	Drug Pka	Initial D/M ratio	DLC	SA-V	SE (wt%)	Drug Mw (g/mol)	Drug TPS (Å)	Drug NHA	Drug LogP	Time (Days)	T=0.25 (FDR)	T=0.5 (FDR)	T=1.0 (FDR)
1	104000	0	282.50	7.76	0.80	0.35	76.73	0	130.08	65.72	5	-0.80	0.00	0.09	0.19	0.35
1	104000	0	282.50	7.76	0.80	0.35	76.73	0	130.08	65.72	5	-0.80	0.26	0.09	0.19	0.35
1	104000	0	282.50	7.76	0.80	0.35	76.73	0	130.08	65.72	5	-0.80	1.06	0.09	0.19	0.35
1	104000	0	282.50	7.76	0.80	0.35	76.73	0	130.08	65.72	5	-0.80	2.09	0.09	0.19	0.35
1	104000	0	282.50	7.76	0.80	0.35	76.73	0	130.08	65.72	5	-0.80	3.11	0.09	0.19	0.35
1	104000	0	282.50	7.76	0.80	0.35	76.73	0	130.08	65.72	5	-0.80	4.13	0.09	0.19	0.35
1	104000	0	282.50	7.76	0.80	0.35	76.73	0	130.08	65.72	5	-0.80	5.08	0.09	0.19	0.35
1	104000	0	282.50	7.76	0.80	0.35	76.73	0	130.08	65.72	5	-0.80	6.10	0.09	0.19	0.35
1	104000	0	282.50	7.76	0.80	0.35	76.73	0	130.08	65.72	5	-0.80	7.17	0.09	0.19	0.35
1	104000	0	282.50	7.76	0.80	0.35	76.73	0	130.08	65.72	5	-0.80	8.12	0.09	0.19	0.35
1	104000	0	282.50	7.76	0.80	0.35	76.73	0	130.08	65.72	5	-0.80	9.13	0.09	0.19	0.35
1	104000	0	282.50	7.76	0.80	0.35	76.73	0	130.08	65.72	5	-0.80	10.14	0.09	0.19	0.35
1	104000	0	282.50	7.76	0.80	0.35	76.73	0	130.08	65.72	5	-0.80	11.09	0.09	0.19	0.35
1	104000	0	282.50	7.76	0.80	0.35	76.73	0	130.08	65.72	5	-0.80	12.16	0.09	0.19	0.35
1	104000	0	282.50	7.76	0.80	0.35	76.73	0	130.08	65.72	5	-0.80	13.11	0.09	0.19	0.35
1	104000	0	282.50	7.76	0.80	0.35	76.73	0	130.08	65.72	5	-0.80	14.12	0.09	0.19	0.35
1	104000	0	282.50	7.76	0.80	0.35	76.73	0	130.08	65.72	5	-0.80	15.19	0.09	0.19	0.35
1	104000	0	282.50	7.76	0.80	0.35	76.73	0	130.08	65.72	5	-0.80	16.14	0.09	0.19	0.35
1	104000	0	282.50	7.76	0.80	0.35	76.73	0	130.08	65.72	5	-0.80	17.34	0.09	0.19	0.35

Supplementary Table 2. Summary of the type and range of hyperparameters screened for the 17-feature MLR and LASSO models. Optimal hyperparameter configurations for models developed using nested cross validation are shown in **bold**.

Hyperparameter	MLR	lasso
"positive"	TRUE; **FALSE**	**TRUE**; FALSE
"alpha"	n/a	**0.25**; 0.5; 1.0

Supplementary Table 3. Summary of the type and range of hyperparameters screened for the 17-feature PLS models. Optimal hyperparameter configurations for models developed using nested cross validation are shown in **bold**.

Hyperparameter	PLS
"n_components"	**2**; 4; 6
"max_iter"	500; 1000

Supplementary Table 4. Summary of the type and range of hyperparameters screened for the 17-feature DT and RF models. Optimal hyperparameter configurations for models developed using nested cross validation are shown in **bold**.

Hyperparameter	DT	RF
"criterion"	**squared_error**; friedman_mse; absolute_error; poisson	**squared_error**; absolute_error;
"splitter"	best; random	n/a
"min_sample_split"	2; **4**; 6	**2**; 4; 6; 8
"min_sample_leaf"	1; 2; **4**	1; **2**; 4
"max_features"	**None**; auto; sqrt; log2	auto; sqrt
"ccp_alpha"	**0**, 0.5, 0.1, 0.15	**0**; 0.05; 0.1
"n_estimators"	n/a	**100**; 200; 300; 400
"bootstrap"	n/a	**TRUE**; FALSE
"oob_score"	n/a	**TRUE**; FALSE

Supplementary Table 5. Summary of the type and range of hyperparameters screened for the 17-feature LightGBM, XGBoost, and NGBoost models. Optimal hyperparameter configurations for models developed using nested cross validation are shown in **bold**.

Hyperparameter	LightGBM	XGBoost	NGBoost
"n_estimators"	100; 150; 200; **250**; 300; 400; 500; 600	100; 150; 300; 400; 500, **600**	100; 200; **300**; 400; 500; 600; 800
"boosting_type"	gbdt; **dart**; goss	gbtree; gblinear; **dart**	n/a
"num_leaves"	**16**; 32; 64; 128; 256	n/a	n/a
"learning_rate"	**0.1**; 0.01; 0.001; 0.0001;	**0.1**; 0.01; 0.001; 0.0001	0.1; **0.01**; 0.001
"min_child_weight"	0.001; **0.01**; 0.1; 1; 10	1; 2; **4**; 5;10	n/a
"subsample"	0.4; 0.6; **0.8**; 1.0	**0.8**; 1.0	n/a
"min_child_samples"	2; 10; 20; **40**; 100	n/a	n/a
"reg_alpha"	**0**; 0.005; 0.01; 0.015	0.001; 0.01; **0.1**; 0.5	n/a
"reg_lambda"	**0**; 0.005; 0.01; 0.015	0.001; 0.01; 0.1; **0.5**	n/a
"max_depth"	n/a	**6**; 15; 30; 40; 60	n/a
"gamma"	n/a	**0**; 2; 4; 6; 8; 10	n/a
"max_delta_step"	n/a	1; 2; 4; **6**; 8; 10	n/a
"minibatch_frac"	n/a	n/a	1; **0.8**; 0.5
"col_sample"	n/a	n/a	1; 0.8; **0.5**
"base_estimator"	n/a	n/a	DecisionTreeRegressor(criterion='squared_error', max_depth= ***X***)<br>***X*** = 2; 4; **8**; 12; 16; or 32

Supplementary Table 6. Summary of the type and range of hyperparameters screened for the 17-feature k-NN model. Optimal hyperparameter configurations for model developed using nested cross validation are shown in **bold**.

Hyperparameter	k-NN
“n_neighbors”	2; 4; 5; 6; 8; 10; 12; 15; 20; 25; **30**; 50
“weights”	uniform; **distance**
“algorithm”	Auto; **ball_tree**; kd_tree; brute
“leaf_size”	10; 30; **50**; 75; 100
“p”	**1**; 2

Supplementary Table 7. Summary of the type and range of hyperparameters screened for the 14-feature SVR model. Optimal hyperparameter configurations for model developed using LOGO cross validation are shown in bold.

Hyperparameter	SVR
“kernal”	linear; poly; **rbf**; sigmoid
“degree*”	**None**; 2; 3; 4; 5; 6
“gamma”	**scale**; auto
“C”	0.1; 0.5; **1**; 2
“epsilon”	0.001; 0.01; 0.1; 0.15; **0.2**
“shrinking”	TRUE; **FALSE**

**poly kernel only*

Supplementary Table 8. Summary of the type and range of hyperparameters screened for the 17-feature NN model. Optimal hyperparameter configurations for model developed using nested cross validation are shown in **bold**.

Hyperparameter	NN
“hidden_layers”	**1**; 2; 3
“neurons”	10; 20; **30**
"learning_rate”	**0.001**; 0.01; 0.1
“regularization”	0.01; 0.001; **0.0001**
“dropout”	0.0; **0.1**; 0.2
“batch_size”	**10**; 20; 40
“activation”	**softmax**; relu; tanh; sigmoid
“optimizer”	rmsprop

Supplementary Table 9: Summary of the results obtained for the LGBM, RF, NGB, and DT **few-shot model (17-feature)** from the inner and outer loops after each nested cross validation trial (n=10). The mean and standard deviation obtained across all trials are also summarized.

Trial #	LGBM		RF		NGB		XGB		DT	
	inner loop	outer loop	inner loop	outer loop	inner loop	outer loop	inner loop	outer loop	inner loop	outer loop
n=1	0.117	0.118	0.128	0.127	0.127	0.125	0.129	0.130	0.159	0.158
n=2	0.127	0.125	0.123	0.129	0.122	0.112	0.124	0.123	0.150	0.142
n=3	0.129	0.118	0.133	0.122	0.131	0.114	0.132	0.142	0.145	0.154
n=4	0.120	0.132	0.128	0.107	0.120	0.142	0.137	0.120	0.158	0.144
n=5	0.120	0.106	0.134	0.111	0.133	0.108	0.129	0.148	0.152	0.166
n=6	0.117	0.131	0.122	0.145	0.136	0.110	0.136	0.113	0.149	0.172
n=7	0.125	0.107	0.121	0.150	0.131	0.100	0.134	0.112	0.167	0.139
n=8	0.131	0.103	0.136	0.104	0.122	0.155	0.140	0.115	0.162	0.129
n=9	0.129	0.099	0.132	0.095	0.130	0.091	0.128	0.165	0.157	0.104
n=10	0.133	0.102	0.144	0.100	0.123	0.163	0.141	0.093	0.145	0.230
Mean	0.125	0.114	0.130	0.119	0.128	0.122	0.133	0.126	0.154	0.154
Stdev	0.006	0.012	0.007	0.019	0.005	0.024	0.006	0.021	0.007	0.033

Supplementary Table 10: Summary of the results obtained for the NN, kNN, SVR, lasso, PLS, and MLR **few-shot model (17-feature)** from the inner and outer loops after each nested cross validation trial (n=10). The m ean and standard deviation obtained across all trials are also summarized.

Trial #	NN		kNN		SVR		lasso		PLS		MLR	
	inner loop	outer loop	inner loop	outer loop	inner loop	outer loop	inner loop	outer loop	inner loop	outer loop	inner loop	outer loop
n=1	0.170	0.160	0.182	0.176	0.204	0.204	0.243	0.246	0.248	0.248	0.247	0.253
n=2	0.163	0.175	0.180	0.188	0.219	0.202	0.248	0.253	0.255	0.253	0.242	0.233
n=3	0.181	0.193	0.173	0.164	0.207	0.184	0.248	0.256	0.258	0.252	0.230	0.240
n=4	0.182	0.169	0.176	0.186	0.196	0.223	0.224	0.232	0.242	0.231	0.249	0.239
n=5	0.170	0.185	0.191	0.159	0.220	0.166	0.246	0.256	0.246	0.222	0.231	0.247
n=6	0.168	0.183	0.192	0.155	0.226	0.160	0.250	0.261	0.237	0.262	0.249	0.225
n=7	0.165	0.180	0.183	0.139	0.209	0.277	0.244	0.261	0.257	0.219	0.244	0.270
n=8	0.167	0.183	0.185	0.128	0.197	0.266	0.236	0.257	0.258	0.219	0.245	0.218
n=9	0.144	0.192	0.170	0.232	0.200	0.362	0.243	0.265	0.224	0.278	0.230	0.279
n=10	0.151	0.220	0.155	0.237	0.181	0.441	0.236	0.259	0.235	0.335	0.276	0.344
Mean	0.166	0.184	0.179	0.176	0.206	0.248	0.242	0.255	0.246	0.252	0.244	0.255
Stdev	0.012	0.016	0.011	0.036	0.013	0.091	0.008	0.009	0.012	0.035	0.013	0.037

Supplementary Table 11: Summary of the results obtained for the LGBM, RF, NGB, and DT **zero-shot model (14-feature)** from the inner and outer loops after each nested cross validation trial (n=10). The mean and standard deviation obtained across all trials are also summarized.

Trial #	LGBM		RF		NGB		XGB		DT	
	inner loop	outer loop	inner loop	outer loop	inner loop	outer loop	inner loop	outer loop	inner loop	outer loop
n=1	0.160	0.149	0.157	0.153	0.154	0.154	0.161	0.163	0.183	0.181
n=2	0.147	0.164	0.175	0.171	0.173	0.169	0.178	0.175	0.199	0.195
n=3	0.174	0.152	0.181	0.187	0.169	0.181	0.182	0.196	0.210	0.226
n=4	0.172	0.208	0.177	0.169	0.160	0.143	0.175	0.195	0.194	0.162
n=5	0.186	0.142	0.163	0.153	0.179	0.153	0.150	0.173	0.204	0.244
n=6	0.163	0.212	0.168	0.182	0.185	0.157	0.176	0.201	0.182	0.231
n=7	0.172	0.228	0.181	0.139	0.190	0.153	0.192	0.147	0.208	0.141
n=8	0.188	0.122	0.155	0.215	0.161	0.199	0.191	0.127	0.226	0.131
n=9	0.196	0.123	0.197	0.112	0.149	0.227	0.197	0.129	0.205	0.313
n=10	0.143	0.295	0.150	0.255	0.168	0.252	0.149	0.324	0.174	0.302
mean	0.170	0.180	0.170	0.174	0.169	0.179	0.175	0.183	0.199	0.213
std	0.017	0.055	0.015	0.040	0.013	0.036	0.017	0.056	0.016	0.063

Supplementary Table 12: Summary of the results obtained for the NN, kNN, SVR, lasso, PLS, and MLR **zero-shot model (14-feature)** from the inner and outer loops after each nested cross validation trial (n=10). The mean and standard deviation obtained across all trials are also summarized.

Trial #	NN		kNN		SVR		lasso		PLS		MLR	
	inner loop	outer loop	inner loop	outer loop	inner loop	outer loop	inner loop	outer loop	inner loop	outer loop	inner loop	outer loop
n=1	0.218	0.216	0.217	0.223	0.229	0.222	0.244	0.244	0.279	0.272	0.276	0.279
n=2	0.205	0.202	0.213	0.205	0.243	0.231	0.263	0.259	0.268	0.281	0.269	0.272
n=3	0.216	0.206	0.215	0.194	0.230	0.248	0.273	0.256	0.268	0.288	0.286	0.290
n=4	0.192	0.214	0.222	0.255	0.233	0.210	0.268	0.287	0.266	0.244	0.268	0.274
n=5	0.196	0.218	0.222	0.257	0.227	0.254	0.269	0.297	0.270	0.247	0.284	0.300
n=6	0.195	0.168	0.202	0.252	0.229	0.199	0.270	0.306	0.268	0.296	0.270	0.288
n=7	0.209	0.238	0.217	0.272	0.228	0.314	0.256	0.295	0.280	0.240	0.292	0.261
n=8	0.210	0.177	0.227	0.145	0.264	0.154	0.256	0.295	0.244	0.290	0.268	0.298
n=9	0.191	0.258	0.241	0.152	0.207	0.329	0.261	0.301	0.249	0.298	0.264	0.317
n=10	0.161	0.312	0.192	0.281	0.193	0.410	0.243	0.292	0.276	0.342	0.324	0.383
mean	0.199	0.221	0.217	0.224	0.228	0.257	0.260	0.283	0.267	0.280	0.280	0.296
std	0.017	0.041	0.013	0.048	0.019	0.075	0.011	0.022	0.012	0.031	0.018	0.035

Supplementary Table 13. Mean absolute error (MAE) values obtained using the different ML algorithms (few-shot versus zero-shot) following prediction of fractional drug release during nested cross-validation (i.e., $n = 10$ trials).

		LGBM	RF	NGB	XGB	DT	NN	k-NN	SVR	lasso	PLS	MLR
**few-shot**	**Inner loop (Validation)**	0.125	0.130	0.128	0.133	0.154	0.166	0.179	0.206	0.242	0.246	0.244
	**Outer loop (Test)**	0.114	0.119	0.122	0.126	0.154	0.184	0.176	0.248	0.255	0.252	0.255
**zero-shot**	**Inner loop (Validation)**	0.170	0.170	0.169	0.175	0.199	0.199	0.217	0.228	0.260	0.267	0.280
	**Outer loop (Test)**	0.180	0.174	0.179	0.183	0.213	0.221	0.224	0.257	0.283	0.280	0.296

Supplementary Table 14. Table summarizing the impact of feature cluster removal (i.e., based on their respective linkage distances) on the predictive performance of the zero-shot RF model. The performance of the RF model with various numbers of input features was assessed by comparing the average and standard deviation of the AE values obtained from a series of trials (*n*=10 trials) that randomly grouped 20% of the drug-polymer combinations as a holdout test set.

No. of features	Mean absolute error (n=10)	Standard Deviation (n=10)	Wards linkage distance
**14**	0.160	0.033	0.00
**13**	0.161	0.032	0.07
**11**	0.161	0.027	0.14
**10**	0.162	0.028	0.29
**9**	0.169	0.030	0.36
**8**	0.175	0.032	0.50
**7**	0.179	0.031	0.64
**5**	0.176	0.035	0.79
**4**	0.217	0.049	0.86
**3**	0.335	0.060	0.93

Supplementary Table 15: Summary of the results obtained from the characterization of the salicylic acid and olapirib-loaded PLGA systems. Including drug encapsulation efficiency (EE), drug loading capacity (DLC), surface area-to-volume ratio (SA-V), and particle size (i.e., mean diameter).

Formulation	EE	DLC	SA-V (Diameter)
**OLA-PLGA**	72.2%	28.9%	74<br>(81.8 µm)
**SA-PLGA**	4.8%	1.6%	138<br>(43.6 µm)
