# Supplementary material for: Machine learning models to accelerate the design of polymeric long-acting injectables
Source: Nat Commun. 2023 Jan 10;14:35. doi: 10.1038/s41467-022-35343-w (PMC9832011; doi:10.1038/s41467-022-35343-w)
Supplement: Supplementary file 3 — Description of Additional Supplementary Files [file 41467_2022_35343_MOESM3_ESM.pdf]

Description of Additional Supplementary Files

Supplementary Data 1: Initial dataset file used for ML model training. Includes 17 inputs features, fractional drug release targets, experimental indexes, and drug-polymer groups.
